# Sun Tzu Art of War-Inspired Technique for the Elusive Sternoclavicular Joint (SCJ) Arthritis: The Badortho Procedure

**DOI:** 10.7759/cureus.105465

**Published:** 2026-03-18

**Authors:** Badrul Akmal Hisham Md Yusoff, Azmi Abdul Latiff, Muhammad Karbela Reza Ramlan, Nik Kamarul Arif Nik Kamaruzman, Mohamad Azwan Aziz

**Affiliations:** 1 Orthopedics and Traumatology, Universiti Kebangsaan Malaysia Medical Centre, Kuala Lumpur, MYS; 2 Orthopedics and Traumatology, Hospital Pakar Pusrawi, Kuala Lumpur, MYS

**Keywords:** biomechanics, distal clavicle resection, mumford procedure, shoulder surgery, sternoclavicular joint arthritis

## Abstract

Sternoclavicular (SC) joint arthritis and instability are uncommon and often diagnostically and therapeutically challenging due to complex anatomy and proximity to vital mediastinal structures. We describe a unique staged management strategy inspired by the Chinese military stratagem Sheng Dong Ji Xi (“feign attack in the east, strike in the west”), as well as a new distal clavicle resection surgical technique, whereby symptomatic functional impairment was ultimately addressed through targeted distal clavicle resection while leaving a chronic painless SC joint dislocation untouched. This case highlights the importance of understanding the anatomy and biomechanics of the skeletal structure and demonstrates a pragmatic strategy for managing complex clavicular joint pathology. We name this novel surgical technique the Badortho sternoclavicular joint (SCJ) procedure. A 37-year-old male motorcyclist sustained anterior sternoclavicular (SC) joint subluxation following trauma. Initial conservative management failed due to persistent instability. Open SC stabilization with anchor sutures failed within eight months, requiring revision reconstruction using gracilis autograft to reconstruct the costoclavicular ligament. The patient initially returned to work; however, he developed worsening pain over the sternoclavicular joint, especially during axial loading of the upper limb. In view of multiple procedures done over SCJ, we decided on a different approach where we reduced the force transfer to the SCJ through lateral clavicle excision. The patient underwent a Badortho SCJ procedure, an open lateral distal clavicle excision of 15 mm, with preservation of stabilizers of the acromioclavicular joint. At two-year follow-up, he achieved a full range of motion and a pain score of zero. Chronic painless anterior SC dislocation persisted but required no further intervention. This case highlights the potential of this new surgical approach, which is less invasive and less demanding to treat complex SCJ arthritis.

## Introduction

Sternoclavicular (SC) joint pathology is rare, accounting for less than 3% of shoulder girdle dislocations, and degenerative arthritis of the SC joint remains underreported and poorly understood [1]. Given the rarity of these injuries, their assessment and management can pose a diagnostic and therapeutic dilemma for the clinician. While the majority of anterior sternoclavicular joint instability resolves with non-operative management, a minority of patients experience persistent, symptomatic instability that ultimately necessitates surgical stabilization. A wide array of surgical techniques for managing sternoclavicular joint (SCJ) pathology has been described in the literature, with no current consensus or high-level evidence establishing the superiority of one approach over another. The following representative techniques illustrate the evolution and diversity of surgical strategies [1]. Ibrahim et al. described a reconstruction technique of SCJ using suture tape [2], Tytherleigh-Strong et al. described a suture repair with internal brace at SCJ [3], Qu et al. used hook plate and locking plate to stabilize the SCJ [4,5], Tytherleigh-Strong et al. used hamstring autograft to reconstruct the SCJ, and Feng et al. used Blazer plate at SCJ [6,7]. All these procedures described at SCJ, which impose an increased risk of major neuromuscular injury, are commonly done in a center where a cardiothoracic surgeon should be on standby and are technically demanding, especially for a young surgeon. Thus, we seek to find a better surgical approach to tackle the SCJ arthritis.

Distal clavicle resection, originally described by Mumford, remains an established surgical treatment when conservative therapy fails in acromioclavicular joint pathology, but has never been described to treat the SC joint pathology [8]. The Chinese military stratagem Sheng Dong Ji Xi (make a sound in the east, then strike in the west) describes a tactical diversion used to achieve strategic objectives.

In orthopedic surgery, symptom-driven intervention may require addressing the true pain generator rather than the most radiographically dramatic pathology. We present a case illustrating this principle in managing chronic SC joint dislocation through arthroscopic distal clavicle resection.

## Case presentation

**Figure 1:**
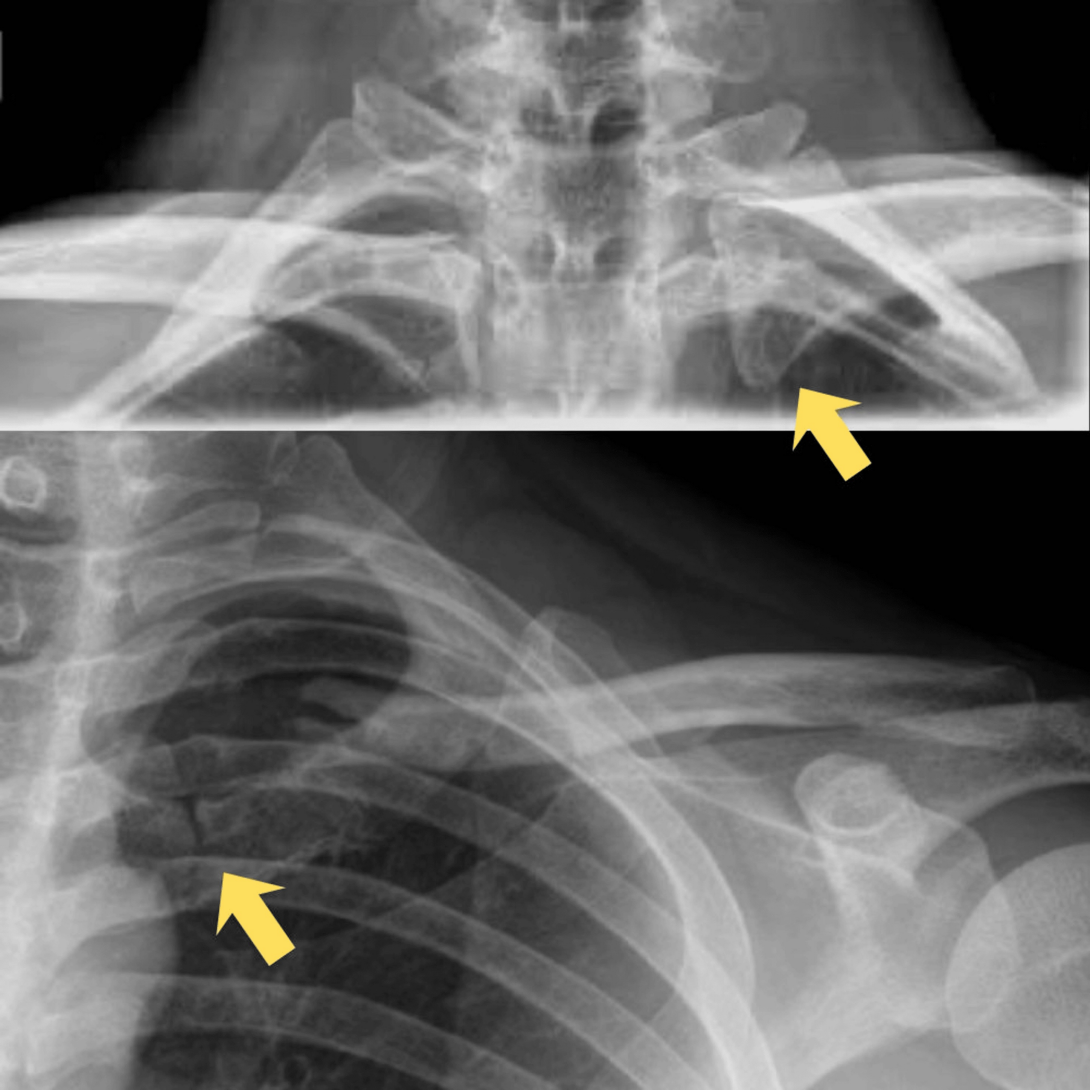
The image shows a serendipity view (above) and an anteroposterior chest X-ray view (below). Yellow arrows show an anteriorly subluxated sternoclavicular joint.

A 37-year-old male motorcyclist presented following a road traffic accident with left shoulder pain and swelling. Initial radiographs demonstrated a closed, undisplaced fracture of the distal clavicle and a fracture of the medial clavicle with anterior SC joint subluxation (Figure 1). Neurovascular examination was normal, and conservative management with an arm sling was initiated.

One-month post-injury, the patient reported persistent SC joint pain and limited shoulder abduction to 90°. Due to symptomatic instability, open SC joint stabilization using anchor sutures in a figure-of-eight configuration was performed four months post-injury.

**Figure 2:**
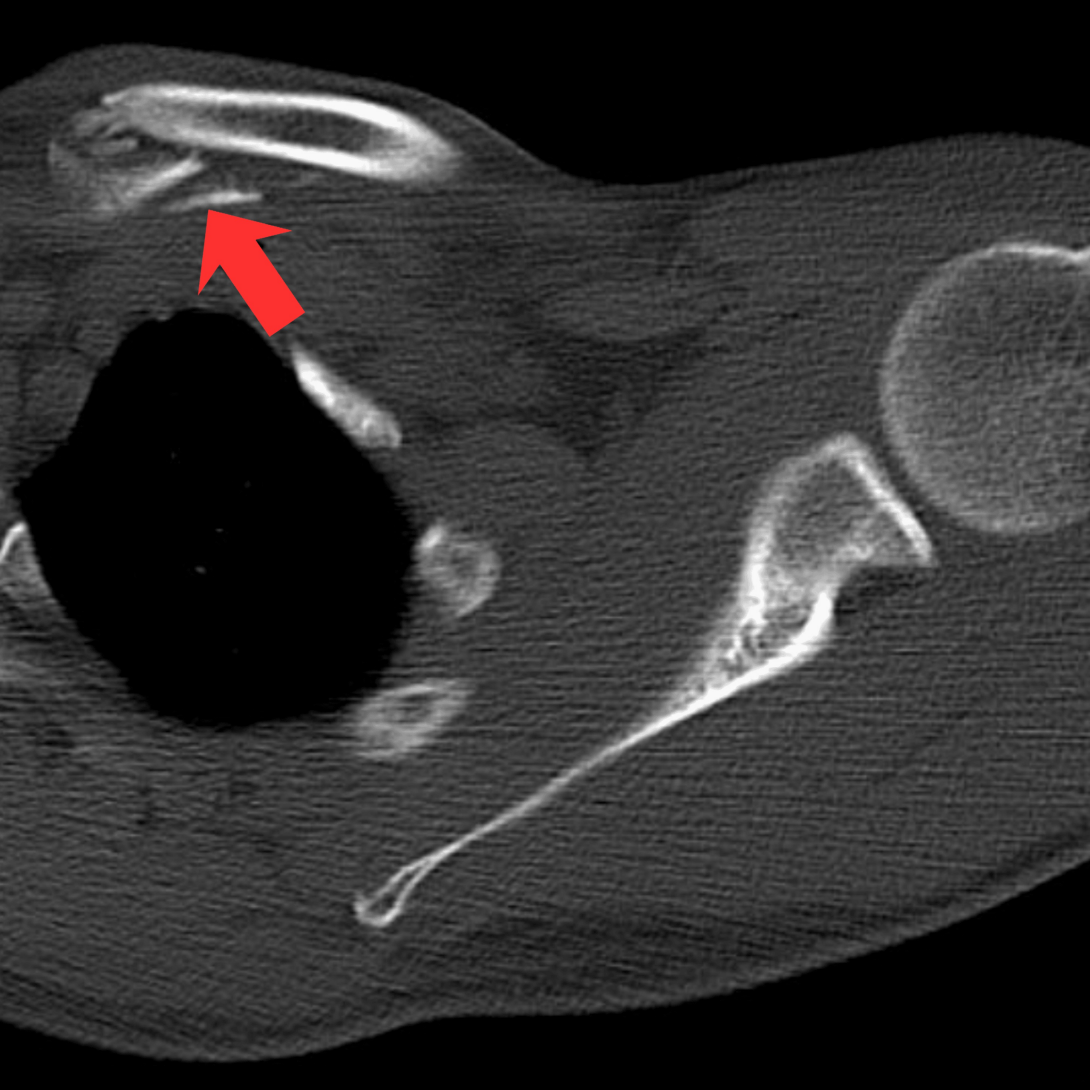
The image shows an axial view of CT scan thorax after failed sternoclavicular joint procedures. Red arrow shows persistent anteriorly subluxated sternoclavicular joint.

Eight months later, fixation failure occurred with recurrent anterior subluxation. Revision reconstruction using a gracilis autograft passed beneath the first rib and through the clavicle to reconstruct the costoclavicular ligament was performed. The patient regained full shoulder motion and returned to work within three months. Two years later, painless recurrent anterior SC joint dislocation occurred without trauma. The patient developed progressive shoulder pain with reduced abduction and forward flexion to 90°, significantly impairing daily activities, with a visual analog score (VAS) of 5-6 on activity [9]. Imaging demonstrated a healed distal clavicle fracture with minimal degenerative AC joint changes on magnetic resonance imaging. Computed tomography confirmed recurrent anterior SC joint subluxation (Figure 2).

**Figure 3:**
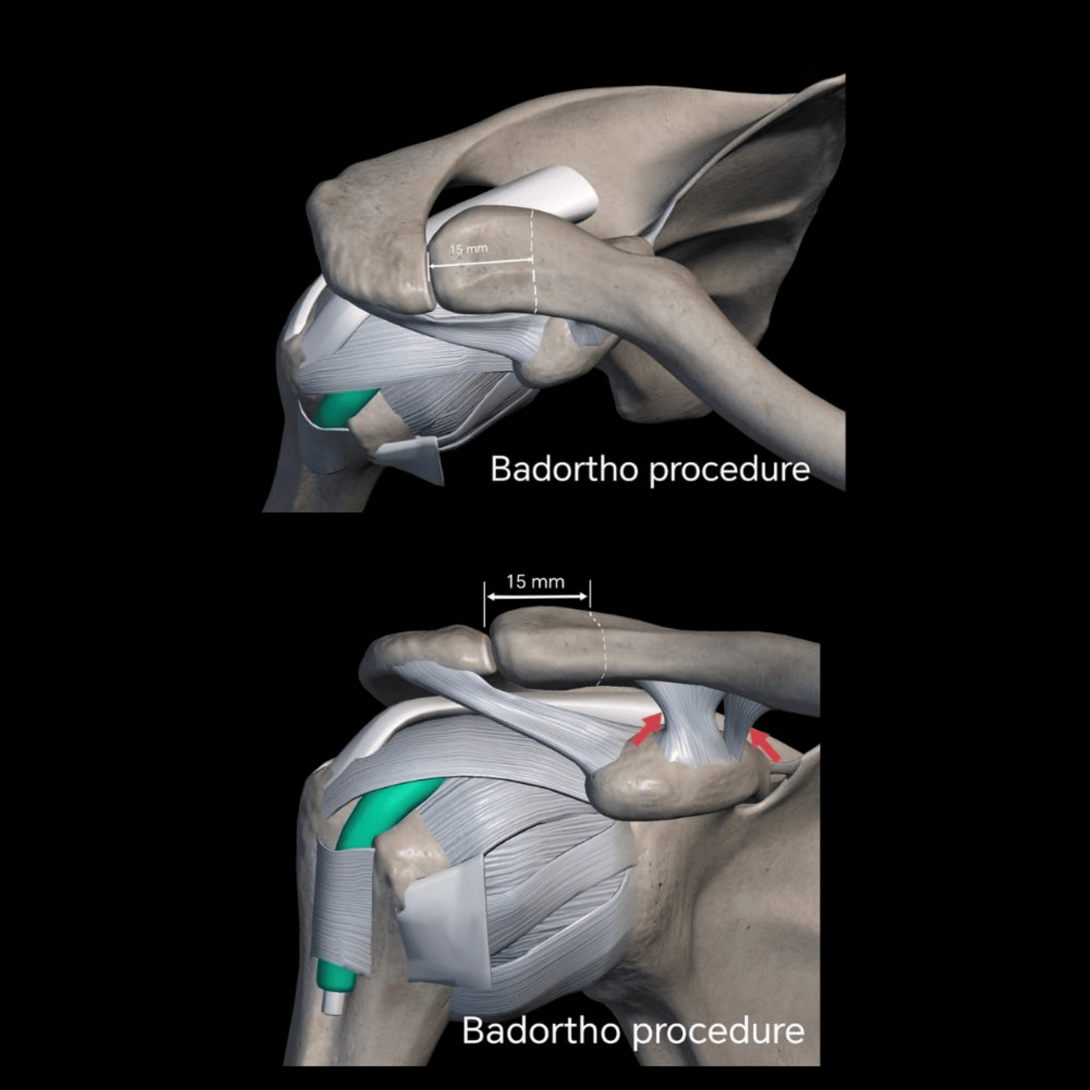
Site of lateral clavicle excision. This image shows the site of excision of the lateral clavicle. The white dots are located 15 mm from the distal end of the lateral clavicle. This image was created by the author of this study using Canva Pro software (Sydney, Australia).

**Figure 4:**
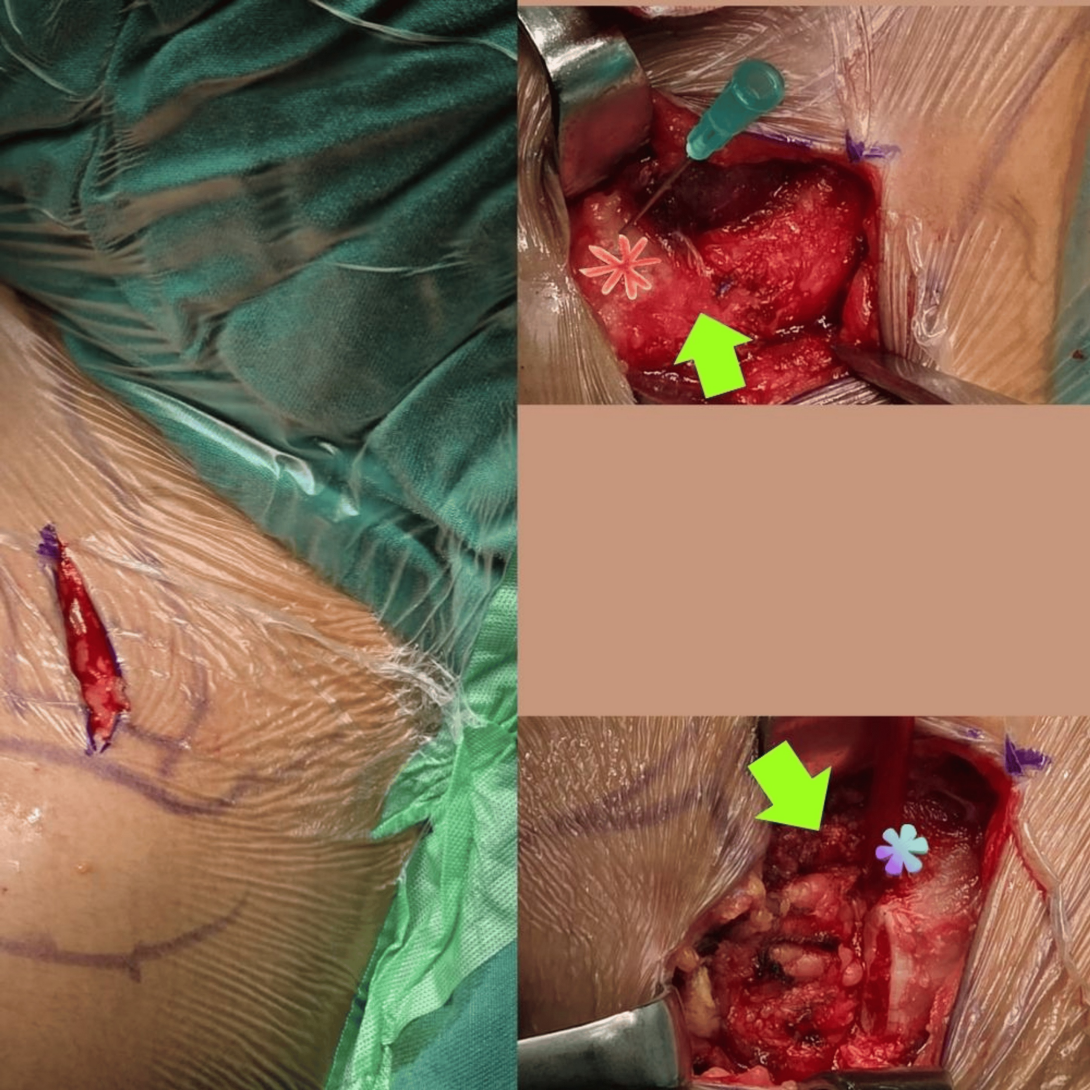
The image shows the intra-operative image of the patient. The patient was positioned supine at a 30° inclined under general anesthesia. The acromioclavicular joint and the coracoid were identified, and skin marking was done. Marcain with adrenalin was infiltrated to reduce subcutaneous bleeding as well as for pain relief post operative. After the skin incision (Figure 4), the wound is opened in layers until the bony surface of the distal clavicle is reached. A hypodermic needle is inserted at the ACJ to identify it. From the needle point which marks the lateral distal clavicle, a 15 mm length was measured. A horizontal incision was made anterior to the marking. The acromioclavicular ligament and coracoclavicular ligament were identified by finger palpation just inferior to the 15-25 mm mark.

After 12 months of failed second SCJ conservative management, using the gracilis tendon, we finally decided on the third and ultimate surgical intervention. An open lateral distal clavicle resection for SCJ (Badortho SCJ procedure) was performed with resection of 15 mm of the distal clavicle under general anesthesia (Figure 3). The patient was in a beach-chair position at a 30° angle under general anesthesia. The acromioclavicular joint and the coracoid were identified, and skin marking was done. Marcain with adrenaline was infiltrated to reduce subcutaneous bleeding as well as for post-operative pain relief. From the tip of the lateral distal clavicle, a 15 mm length was measured. A horizontal incision was made anterior to the marking. Then, the acromioclavicular ligament and coracoclavicular ligament were identified by finger palpation just inferior to the 15-25 mm mark. These ligaments were preserved during the lateral clavicle excision (Figure 4). The procedure was successfully completed within 30 min.

**Figure 5:**
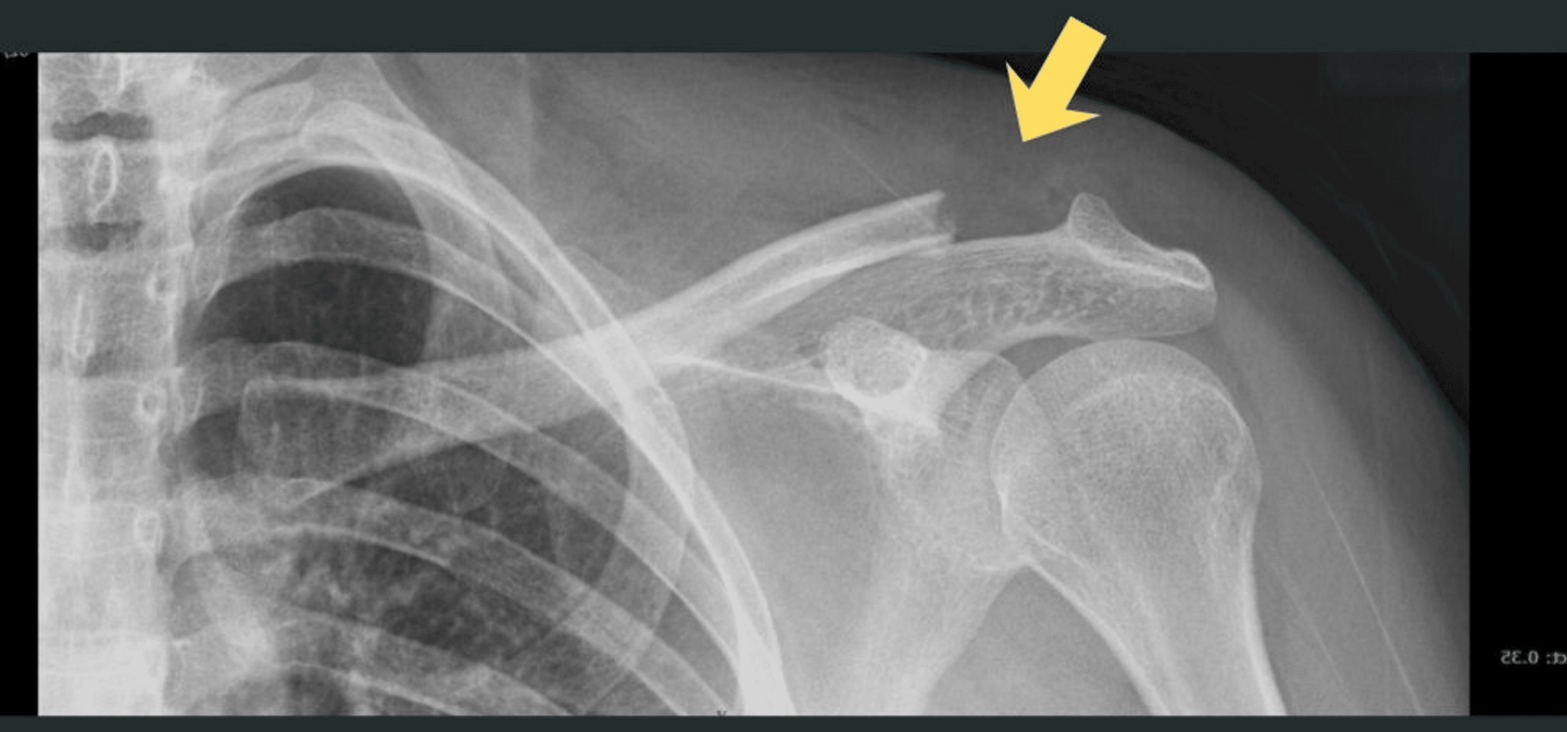
Post-operative X-ray of left clavicle. Post-operative X-ray of the left clavicle in AP view showed no superior migration of the lateral distal clavicle (yellow arrow).

Post-operative rehabilitation commenced on day one with sling protection for two weeks. Post-operative X-ray of the left clavicle showed no superior migration of the distal clavicle, indicating an intact static stabilizer (Figure 5). The patient returned to normal activity at six weeks. Shoulder function was assessed using the Rockwood score, a validated instrument for measuring functional outcomes after SCJ surgery, which comprises pain, range of motion, strength, limitation, and subjective results [10]. The score was measured as 7 pre-operatively, improving to 12 at three months, 13 at six months, 14 at one year, and 14 at two years post-operatively. At the two-year follow-up, the patient had full shoulder range of motion and a visual analog scale (VAS) pain score of 0 [9].

## Discussion

This case demonstrates the indirect biomechanical effect of lateral clavicle resection on symptomatic SC joint pathology. This modified lateral clavicle resection differs from the original Mumford procedure, where the original procedure described the excision of lateral clavicle of 25 mm for acromioclavicular joint pathology. The initial advocates of the procedure advised a minimum resection of 15 mm [11,12]. In a biomechanical investigation into the consequences of clavicular resection, TP branch established that a resection of 5 mm is sufficient to prevent bone-to-bone contact [13]. In contrast, resections greater than 15 mm risk damaging the acromioclavicular and coracoclavicular ligamentous structures, thereby compromising the stability of the acromioclavicular joint and adversely affecting functional outcomes.

Despite dramatic SC joint instability and multiple failed reconstructions, symptom resolution was achieved without further SC joint intervention, reflecting the Sheng Dong Ji Xi principle diverting focus from the apparent lesion to target the true functional pain generator. Interestingly, there were multiple surgical techniques described in the literature for management of SCJ arthritis using clavicle excision [14-17]. Resection of the medial clavicle ranges from 0.5 to 4 cm. Some procedures also required the removal of osteophytes. However, to address the risk of bone regrowth from surface friction, Meis et al. took a more comprehensive approach, combining the resection with an interposition arthroplasty that transposed the sternocleidomastoid muscle [18]. Surgical access was achieved either openly or arthroscopically. Approaching medial clavicle is technically demanding, requires experience, and with increased risk of injuring major branch of aorta and main neurovascular bundle [19].

**Figure 6:**
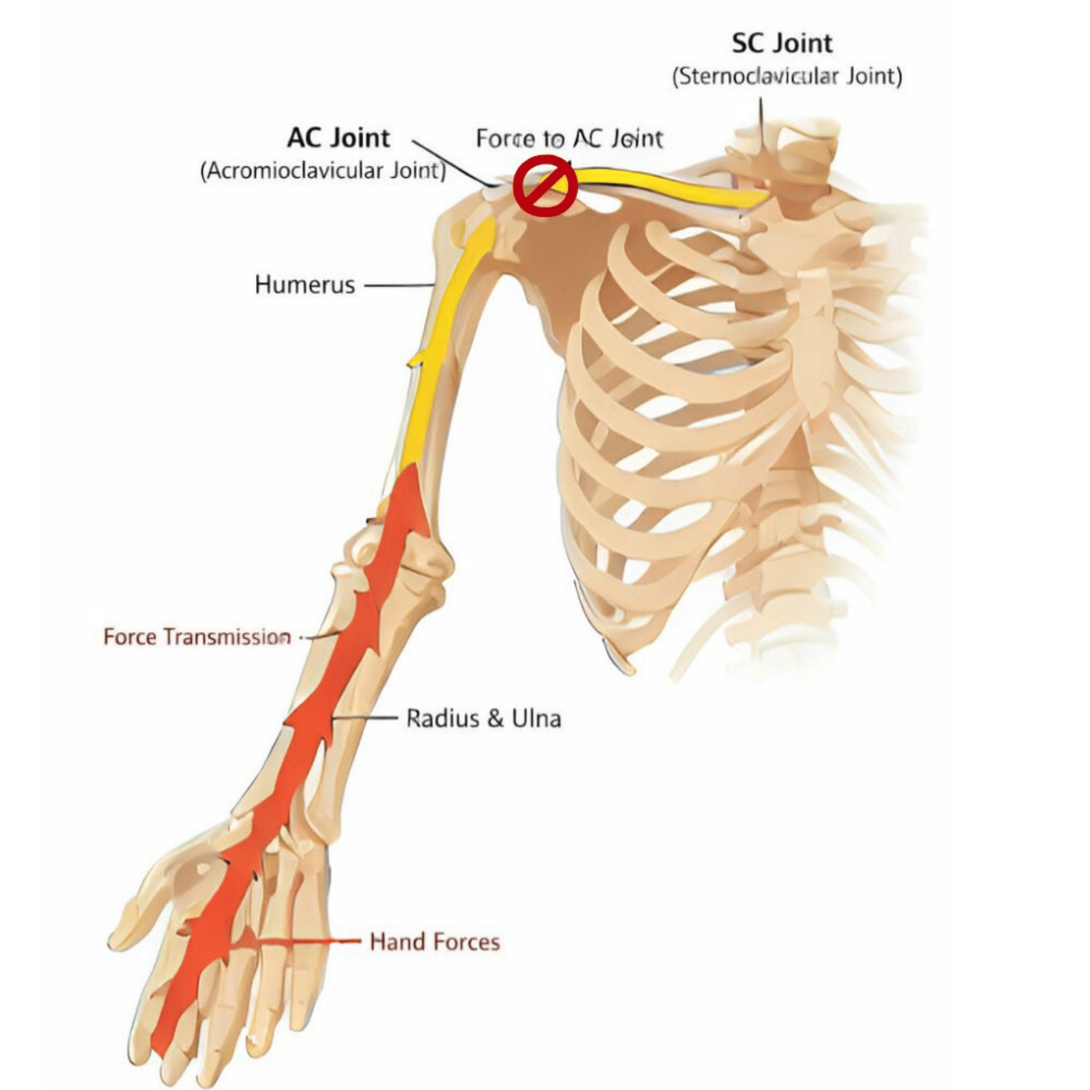
Force of transfer to sternoclavicular joint. The image shows the transfer of force from hand to the sternoclavicular joint during the loading of upper limb. Once resection is performed, the transfer of force to the sternoclavicular joint will be blocked. This image was created by the author of this study using Canva Pro software (Sydney, Australia).

Instead of approaching the medial clavicle, this article described a less technical procedure by approaching the lateral clavicle. The clinical efficacy of distal clavicle resection in alleviating pain originating from the sternoclavicular (SC) joint can be explained through a biomechanical theory of load redistribution, wherein the lateral procedure indirectly decompresses the medial articulation by altering the force transmission dynamics along the clavicular strut. The clavicle functions as a rigid, S-shaped strut that transmits forces from the upper limb to the axial skeleton, with its S-shaped morphology making its biomechanical behavior distinct from that of a straight long bone (Figure 6). When a compressive or axial load is applied from the lateral aspect toward the axial skeleton, the clavicle must absorb and transmit these forces across both the acromioclavicular (AC) and SC joints [20].

The foundational evidence for this unloading mechanism is provided by the biomechanical investigation of Katthagen et al., who demonstrated that force transmission through the SC joint can be surgically modulated by osseous resection [21]. In their controlled cadaveric study, an 80-N axial load applied to the lateral clavicle was used to measure force transmission at the medial SC joint. The results confirmed that resection of the medial clavicle significantly decompressed the SC joint, with a 5-mm resection reducing the transmitted force by a mean of 76.7 N [21]. This confirms that the forces generated at the lateral clavicle are directly transmitted medially, and that removing bone, even at the opposite end, can effectively unload the SC articulation.

Further supporting the concept of the clavicle as an integrated mechanical unit, the distal clavicle and its capsular ligaments are critical for maintaining the strut function that guides scapular and clavicular rotation. The integrity of the AC capsule ensures physiological centering of the joint under rotational loading; when these structures are compromised, there is a significant increase in the amplitude of clavicular motion [22]. Therefore, a distal clavicle resection not only shortens the overall length of this strut but also likely dampens the efficient transfer of rotational and translational energy along the bone. By reducing the lever arm and disrupting the transmission of force at the lateral end, the procedure effectively stress-shields the medial joint, lowering the compressive and shear forces across the arthritic SC joint below the threshold that provokes pain. This mechanism explains how addressing the lateral pathology can provide symptomatic relief at the medial joint, as observed in the presented clinical case.

## Conclusions

This case report introduces a novel application of the arthroscopic distal clavicle resection, traditionally used for acromioclavicular joint pathology, as a potential surgical option for managing symptomatic sternoclavicular joint arthritis. Drawing on the conceptual framework of the Sheng Dong Ji Xi principle, the procedure offered symptomatic relief in a single patient with complex, recurrent SCJ pathology, despite persistent anatomical abnormalities at the medial joint. While the clinical outcome was favorable, the findings should be interpreted with caution. As a single case report, the generalizability of this approach is limited, and the proposed biomechanical mechanism remains theoretical. Further research, including biomechanical studies and larger clinical series, is necessary to validate the efficacy, safety, and indications of this technique before it can be widely recommended.

## References

[1] Morell DJ, Thyagarajan DS (2016). Sternoclavicular joint dislocation and its management: a review of the literature. World J Orthop.

[2] Ibrahim MA, Elsherbiny EA, Farag GA, Nematallah SA (2023). Is reconstructing anterior sternoclavicular joint dislocation by a high-strength tape suture a good choice?. J Musculoskelet Surg Res.

[3] Tytherleigh-Strong G, Pecheva M, Titchener A (2018). Treatment of first-time traumatic anterior dislocation of the sternoclavicular joint with surgical repair of the anterior capsule augmented with internal bracing. Orthop J Sports Med.

[4] Qu YZ, Xia T, Liu GH, Zhou W, Mi BB, Liu J, Guo XD (2019). Treatment of anterior sternoclavicular joint dislocation with acromioclavicular joint hook plate. Orthop Surg.

[5] Qu Y, Xie X, Zhou W (2022). Operative treatment outcomes of anterior sternoclavicular joint dislocation using two experimental methods - an acromioclavicular joint hook plate versus a locking plate: a retrospective study. BMC Musculoskelet Disord.

[6] Tytherleigh-Strong G, Sabharwal S, Peryt A (2022). Clinical outcomes and return to sports after open reduction and hamstring tendon autograft reconstruction in patients with acute traumatic first-time posterior dislocation of the sternoclavicular joint. Am J Sports Med.

[7] Feng D, Yang Y, Kang X, Heng L, Zhang J, Zhu Y (2023). Extra-articular locking plate and trans-articular clavicle hook plate for displaced medial clavicle fractures. Injury.

[8] Mumford EB (1941). Acromioclavicular dislocation: a new operative treatment. J Bone Joint Surg.

[9] Huskisson EC (1974). Measurement of pain. Lancet.

[10] Rockwood CA, Groh GI, Wirth MA, Grassi FA (1997). Resection arthroplasty of the sternoclavicular joint. J Bone Joint Surg Am.

[11] Flatow EL, Cordasco FA, Bigliani LU (1992). Arthroscopic resection of the outer end of the clavicle from a superior approach: a critical, quantitative, radiographic assessment of bone removal. Arthroscopy.

[12] Tolin BS, Snyder SJ (1993). Our technique for the arthroscopic Mumford procedure. Orthop Clin North Am.

[13] Branch TP, Burdette HL, Shahriari AS, Carter FM, Hutton WC (1996). The role of the acromioclavicular ligaments and the effect of distal clavicle resection. Am J Sports Med.

[14] Tytherleigh-Strong G, Griffith D (2013). Arthroscopic excision of the sternoclavicular joint for the treatment of sternoclavicular osteoarthritis. Arthroscopy.

[15] Tytherleigh-Strong G, Gill J, Mulligan A, Al-Hadithy N (2020). Arthroscopic excision arthroplasty of the sternoclavicular joint for osteoarthritis: a case series of 50 patients. Arthroscopy.

[16] Dekker TJ, Lacheta L, Goldenberg BT, Horan MP, Pogorzelski J, Millett PJ (2020). Minimum 5-year outcomes and return to sports after resection arthroplasty for the treatment of sternoclavicular osteoarthritis. Am J Sports Med.

[17] Pingsmann A, Patsalis T, Michiels I (2002). Resection arthroplasty of the sternoclavicular joint for the treatment of primary degenerative sternoclavicular arthritis. J Bone Joint Surg Br.

[18] Meis RC, Love RB, Keene JS, Orwin JF (2006). Operative treatment of the painful sternoclavicular joint: a new technique using interpositional arthroplasty. J Shoulder Elbow Surg.

[19] Nabergoj M, Lädermann A, Chong X, Wang S, Ho SW (2021). Reconstruction of the sternoclavicular joint after excessive medial clavicle resection. Arthrosc Tech.

[20] Hoogervorst P, Bolsterlee B, Pijper M, Aalsma A, Verdonschot N (2019). Forces acting on the clavicle during shoulder abduction, forward humeral flexion and activities of daily living. Clin Biomech.

[21] Katthagen JC, Marchetti DC, Dahl KD, Turnbull TL, Millett PJ (2016). Biomechanical comparison of surgical techniques for resection arthroplasty of the sternoclavicular joint. Am J Sports Med.

[22] Berthold DP, Muench LN, Dyrna F (2022). Current concepts in acromioclavicular joint (AC) instability - a proposed treatment algorithm for acute and chronic AC-joint surgery. BMC Musculoskelet Disord.

